# Knowledge, attitudes, and behavioral intentions of elderly individuals regarding advance care planning: Questionnaire development and testing

**DOI:** 10.1371/journal.pone.0272351

**Published:** 2022-07-28

**Authors:** Hui-Chuan Cheng, Li-Shan Ke, Su-Yu Chang, Hsiu-Ying Huang, Yu-Chen Ku, Ming-Ju Lee

**Affiliations:** 1 School of Nursing, National Taipei University of Nursing and Health Sciences, Taipei, Taiwan; 2 Department of Nursing, Taipei Veterans General Hospital, Taipei, Taiwan; 3 Long-Term Care, Taipei Municipal Gan-Dau Hospital, Taipei, Taiwan; Al-Ahliyya Amman University, JORDAN

## Abstract

**Background:**

Studies have indicated that the advance care planning knowledge and attitudes of elderly individuals strongly affect their implementation of advance care planning. A measurement with a theoretical base for evaluating elderly individuals’ knowledge, attitudes, and behavioral intentions regarding advance care planning is lacking.

**Objectives:**

To develop a questionnaire and understand elderly individuals’ knowledge, attitudes, and behavioral intentions regarding implementing advance care planning.

**Methods:**

A cross-sectional questionnaire survey was conducted. The content validity index, and statistical methods, including discrimination, factor, and reliability analysis, were adopted for psychometric testing. Descriptive statistics mainly presented data analysis.

**Results:**

401 elderly individuals were recruited from a medical center and one senior activity center. The content validity index was approximately 0.71–0.92 for the developed questionnaires, the Kuder–Richardson formula 20 was 0.84 for advance care planning knowledge, and the Cronbach’s alpha was 0.86, 0.94, 0.76, and 0.92 for attitudes, behavioral intentions, influencing factors, and subjective norms, respectively. The average score for advance care planning knowledge for elderly individuals was 4.42, with a correct answer rate of 49.1%. They lacked knowledge of advance care planning-related legislation. The mean score for attitudes and behavioral intentions was 14.32 and 3.48, respectively. Elderly individuals agreed that advance care planning has benefits but were worried about the emotional distress caused by advance care planning discussions. Elderly individuals with positive behavioral intentions tend to implement advance care planning. Spouses, children, doctors, and nurses are significant reference people for elderly individuals.

**Conclusions:**

The developed questionnaire exhibits good validity and reliability for understanding elderly individuals’ knowledge, attitudes, and behavioral intentions concerning advance care planning. Advance care planning materials or decision aids suitable for elderly individuals must be developed to increase their understanding of advance care planning. Additionally, the role of nurses is indispensable in promoting advance care planning among elderly individuals.

## Introduction

The purpose of advance care planning (ACP) is to ensure that individuals have a good death at the end of life in accord with their wishes. ACP is the process through which an individual discusses a future care plan with health-care professionals, family members, or significant others [1]. Respecting the hospice right of terminally ill patients, Taiwan implemented the Hospice Palliative Care Act in 2000 [2]. Terminally ill patients can choose to receive life-sustaining treatments or palliative care by signing a *Palliative Care or Life-Sustaining Treatment Intention Letter*. Patients can also appoint persons as health-care agents by signing a *Health-Care Power of Attorney Form*. These are the two most common forms of advance directives (ADs) in Taiwan. Additionally, AD signatories may register their wishes on their National Health Insurance certificate (NHI card), which can consequently be read by health-care institutions [2]. In 2019, Taiwan officially implemented the Patient Right to Autonomy Act. This Act allows an individual aged >20 years and who has full capacity to write an *advance decision* through *ACP consultation*, which is a legal process, to accept or refuse life-sustaining treatments, including artificial nutrition and hydration [3]. The aforementioned two Acts are milestones for respecting patient choice in Taiwan.

The elderly population in Taiwan is increasing dramatically and is poised to increase from 16.1% in 2020 to 45.4% in 2070 [4]. This highlights the need to confront the end-of-life care issues faced by the elderly people in Taiwan. Elderly individuals have unique characteristics, such as poor vision, hearing loss, delayed expression, and even cognitive impairment; therefore, they are a high-risk group prone to losing their decision-making ability [5], and thus, ACP should be implemented before their decision-making ability is lost. However, the number of elderly individuals implementing ACP is low. A Chinese study investigating 900 community-dwelling elderly individuals’ preferences for ACP revealed that the awareness of these individuals regarding “planning ahead” was low [6]. Many studies have indicated that the ACP knowledge and attitudes of elderly individuals strongly affect their ACP implementation [7, 8].

A systematic review study found that most questionnaire surveys on ACP knowledge mainly focused on health-care professionals, whereas only a few focused on elderly individuals [9]. Although Taiwan has been promoting palliative care and patient autonomy for several years, many elderly individuals still have never heard of ACP [10]. A measurement with a theoretical base for evaluating elderly individuals’ knowledge, attitudes, and behavioral intentions concerning ACP is lacking. Moreover, no localized tool is available in Taiwan for measuring the knowledge, attitudes, and behavioral intentions of elderly individuals toward ACP. This prompted the authors to develop the ACP questionnaire based on the theory of reasoned action (TRA). According to the TRA, individuals’ behavioral intentions determine their actual behavior. Attitudes toward the behavior and subjective norms determine behavioral intentions, and individuals’ knowledge contributes to their attitudes [11]. Thus, the study aimed to develop the ACP questionnaire and understand elderly individuals’ knowledge, attitudes, and behavioral intentions regarding implementing ACP.

## Methods

### Study design

A cross-sectional questionnaire survey was conducted. The study period was from May 2021 to April 2022.

### Participants

The participants included elderly patients and their elderly caregivers from the cardiology and hospitalist’s ward in a medical center in Taipei and elderly individuals from a senior activity center. The elderly individuals were required to be aged >65 years and would be excluded if they had severe visual and hearing impairments and could not communicate in Chinese or Taiwanese.

### Measurements

The authors developed the first draft of this questionnaire after reviewing the literature [12–15]. The developed questionnaire inquires into demographic characteristics and knowledge, attitudes, behavior intentions, subjective norms, and influencing factors regarding ACP (see S1 File). The demographic characteristics inquired into are gender, age, education level, marital status, and religious belief. Six experts specializing in geriatric medicine, geriatric nursing, and palliative care were invited to evaluate the questionnaire. The content validity index (CVI) was adopted. The CVI was 0.81, 0.85, 0.81, 0.92, and 0.71 for ACP knowledge, attitudes, behavioral intentions, influencing factors, and subjective norms, respectively. In addition, face validity was assessed before formally recruiting the study participants. Five elderly individuals were invited to read and answer the questionnaire, and they mentioned that the content was understandable. The psychometrics of each part of the questionnaire are detailed as follows:

1. Knowledge of ACP: The knowledge portion involves patient autonomy, ACP, ADs, and Acts in Taiwan. This part of the questionnaire has nine questions, and the scoring system scores for a correct answer (1 point), wrong answer (0 points), and unknown answer (0 points). The correct answers to questions 6, 7, and 9 are “no,” and the remaining answers are “yes.” Higher scores indicate greater knowledge of ACP. The highest 30% of the total knowledge score was established as the high-grade group, and the lowest 30% was the low-grade group. The independent *t* test was used to conduct the discrimination analysis. The nine items exhibited good discrimination (Table 1). Moreover, each item had a significant correlation with the total score (Table 2), and the Kuder–Richardson formula 20 was 0.84.
2. Attitude toward behavior (AB): Attitude measures elderly individuals’ feelings concerning ACP. This part has 12 questions; each question evaluates both belief (Bi) and evaluation (Ei). The scoring system applies a 5-point Likert scale, with scores ranging from “strongly disagree and very unimportant” (1 point) to “strongly agree and very important” (5 points). The higher the total score of attitude is (AB = Bi × Ei), the more positive the attitude of elderly individuals is toward ACP. The construct validity of the “belief” part was tested using the Bartlett test of sphericity (BT) and Kaiser–Mayer–Olkin test (KMO). The BT value was 4264.916 (*p* < 0.001), and the KMO was 0.924, which indicated that it was suitable for factor analysis. Principal component factor analysis (varimax rotation) was used, with a factor loading of 0.5 as the cutoff point (Table 3). Finally, two factors were extracted from the belief scale and named “benefits of ACP” and “burdens of ACP,” respectively. The Cronbach’s alpha was 0.96 for benefits of ACP (seven items) and 0.85 for burdens of ACP (five items). The Cronbach’s alpha was 0.91 for the Bi scale and 0.74 for the Ei scale. The Cronbach’s alpha was 0.86 for attitudes.
3. Behavioral intentions for ACP: This part has six questions that concern discussing ACP with health-care professionals or family members, participating in ACP consultation, and signing ADs, such as the *Palliative Care or Life-Sustaining Treatment Intention Letter* or *advance decision*. The scoring system ranges from “very unlikely” (1 point) to “very likely” (5 points). The Cronbach’s alpha was 0.94 for this part.
4. Factors affecting ACP: This part has eight questions concerning these factors. Responses to these questions are scored using the Likert scale, with scores ranging from “strongly disagree” (1 point) to “strongly agree” (5 points). The Cronbach’s alpha was 0.76.
5. Subjective norm: Two components influence subjective norm: (1) an individual’s belief that vital reference people will adopt a certain behavior (normative belief; NBj) and (2) an individual’s motivation to follow the reference people (motivation to comply; MCj). This part has seven questions. Reference people include spouses, siblings, children, grandchildren, doctors, nurses, and friends. The scoring system consists of a 5-point Likert scale, with scores ranging from “strongly disagree” (1 point) to “strongly agree” (5 points), and “very unwilling” (1 point) to “very willing” (5 points). The subjective norm is NBj × MCj, and the range is 1–25 points. A higher score indicates that an individual is more easily influenced by others. The Cronbach’s alpha was 0.92.

**Table 1:** Discrimination analysis of items in ACP knowledge.

Variables	Mean (SD)	*t*	*p*
**Item 1**		-12.690	< 0.001
Low-grade group	0.44 (0.50)		
High-grade group	0.99 (0.10)		
**Item 2**		-12.527	< 0.001
Low-grade group	0.45 (0.50)		
High-grade group	0.99 (0.07)		
**Item 3**		-33.144	< 0.001
Low-grade group	0.10 (0.31)		
High-grade group	0.99 (0.07)		
**Item 4**		-34.031	< 0.001
Low-grade group	0.10 (0.31)		
High-grade group	1.00 (0.00)		
**Item 5**		-32.582	< 0.001
Low-grade group	0.07 (0.26)		
High-grade group	0.96 (0.20)		
**Item 6**		-4.320	< 0.001
Low-grade group	0.01 (0.09)		
High-grade group	0.11 (0.32)		
**Item 7**		-5.973	< 0.001
Low-grade group	0.01 (0.09)		
High-grade group	0.18 (0.39)		
**Item 8**		-31.186	< 0.001
Low-grade group	0.04 (0.19)		
High-grade group	0.90 (0.30)		
**Item 9**		-12.981	< 0.001
Low-grade group	0.01 (0.12)		
High-grade group	0.51 (0.50)		

**Table 2:** The correlation between the total score and each item in ACP knowledge.

	TK	Item 1	Item 2	Item 3	Item 4	Item 5	Item 6	Item 7	Item 8	Item 9
**TK**	1	0.701**	0.702**	0.818**	0.845**	0.772**	0.292**	0.376**	0.727**	0.573**

Abbreviation: TK, total score of ACP knowledge.

**p< 0.01.

**Table 3:** Factor loading of the items in the ACP attitudes.

Labeling	Item	Factor loading	
		Factor 1	Factor 2
**1**	I believe that patients should be adequately informed about their condition so that they can prepare for the future.	.740	-.247
**2**	I believe that discussing advance care planning will inform my family or friends of my wishes and encourage them to follow them.	.896	-.159
**3**	I believe that discussing advance care planning can reduce the burden of care on my family or friends.	.922	-.155
**4**	I believe that discussing advance care planning with my family or friends will reduce unnecessary pain at the end of my life.	.932	-.185
**5**	I believe that discussing advance care planning with my family or friends will help maintain my quality of life.	.915	-.153
**6**	I believe that discussing advance care planning will upset my doctor.	-.117	.540
**7**	I believe that discussing advance care planning with family or friends will reduce the burden of making medical decisions for me in the future.	.850	-.211
**8**	I believe that discussing advance care planning with my family or friends will reduce conflict when they make medical decisions on my behalf in the future.	.840	-.219
**9**	I believe that discussing advance care planning with my family or friends will make them uncomfortable.	-.071	.718
**10**	I believe that discussing advance care planning will upset me.	-.168	.874
**11**	I believe that discussing advance care planning will bring me bad luck.	-.242	.870
**12**	I believe that discussing advance care planning will make me feel hopeless.	-.279	.827

### Ethical consideration

This study was reviewed by the research site’s institutional review board (IRB). The IRB number is 2021-05-005C. Participants were informed of the study’s aim, the right to withdraw from the study, and their confidentiality and anonymity would be maintained. Written informed consent from participants was obtained.

### Procedures

The authors explained the inclusion criteria to the health-care professionals in the study units and requested them to refer eligible cases. Two research assistants underwent training before data collection. After obtaining the informed consent of study participants, structured interviews were conducted for approximately 20–30 min in a single room or conference room to collect data. Participants would receive an interview fee of NT$200 (equal to ~US$7) after the interview.

### Statistical analysis

The data were analyzed using the SPSS 20.0 software package. The statistical methods for psychometric testing of questionnaires are described in the Methods section. Descriptive statistics, such as mean and standard deviation, were used to describe the variables in the questionnaire.

To calculate the sample size of descriptive research, in the past, 3–10 samples were required for a rough estimate of one item of a questionnaire. Some scholars have proposed that the internal consistency of the available scales can be used to estimate the sample size. If the consistency is moderate, at least 200 samples are required [16]. In psychology and psychiatry, at least 300 samples are recommended [17]. There are 42 questions in this research questionnaire. After considering the above, the research was expected to receive at least 350 elderly individuals.

## Results

### Demographic characteristics

Four hundred ten questionnaires were delivered, of which 401 were completed and responses, with a response rate of 97.8%. A total of 401 elderly individuals (average age: 75.79 years) were included in the study. Of them, 262 were elderly patients; 86 were elderly caregivers from the cardiology and hospitalist’s wards at a medical center, and 53 were elderly individuals from a senior activity center. Of the study participants, 124 (31.0%) had completed elementary school education, 54 (13.5%) had junior high school education, 86 (21.5%) had senior high school education, 85 were college graduates (21.3%), and 13 had completed a Master’s degree and above (3.3%). Regarding marital status, 64.8% of the elderly individuals were married, and 22.7% were widowed. Regarding religious beliefs, 17.9% of the elderly individuals had no religious beliefs, 46.9% believed in Buddhism, 23.2% believed in Taoism or folk beliefs, 8.1% were Christian, and 3.3% were Catholic (Table 4).

**Table 4:** Demographic characteristics (*N* = 401).

Variables	*n* (%)
**Age means (SD)**	75.79 (7.97)
**Participant sources**	
Cardiology	
Elderly patients	133 (33.2)
Elderly caregivers	47 (11.7)
Hospitalist’s ward	
Elderly patients	129 (32.2)
Elderly caregivers	39 (9.7)
Senior activity center	
Elderly individuals	53 (13.2)
**Gender**	
Male	220 (54.9)
Female	181 (45.1)
**Education level**	
Illiterate	29 (7.2)
Literacy	8 (2.0)
Elementary school	124 (31.0)
Junior high school	54 (13.5)
Senior high school	86 (21.5)
College degree	85 (21.3)
Master degree and above	13 (3.3)
Other	1 (0.3)
**Marital status**	
Unmarried	22 (5.5)
Married	260 (64.8)
Divorced	26 (6.5)
Widowed	91 (22.7)
Separate	1 (0.2)
Cohabit	1 (0.2)
**Religion**	
None	71 (17.9)
Buddhism	186 (46.9)
Taoism	92 (23.2)
Christian	32 (8.1)
Catholic	13 (3.3)
Other	3 (0.8)

### Knowledge of ACP

The average score for ACP knowledge was 4.42 (SD = 2.60, range 1–9) points, with a correct answer rate of 49.1%. The three questions with the highest correct answer rate were “When approaching the end of life, everyone has the right to accept or refuse any treatment” (78.8%), “When approaching the end of life, everyone has the right to accept or refuse any examination” (78.1%), and “Signing the *Palliative Care or Life-Sustaining Treatment Intention Letter* indicates that the terminally ill patient can still receive medical care but no longer receive meaningless tests and treatments, such as intubation or a respirator” (66.8%). This result demonstrated that elderly individuals are aware of patient rights and understand that signing ADs will not affect their medical care.

The three questions with the lowest correct answer rate were “The health-care power of attorney designee must be a spouse or blood relative” (6.0%), “Individuals must sign an advance decision after implementing ACP consultation” (10.7%), and “Once an advance directive is signed, it cannot be changed or withdrawn” (28.4%; Table 5). This result revealed that elderly individuals are unclear about the current ACP-related legislation in Taiwan.

**Table 5:** Knowledge of advance care planning (*N* = 401).

Labeling	Item	Answered Correctly	Answered Incorrectly	Ranking
		*n* (%)	*n* (%)	
**1**	When approaching the end of life, everyone has the right to accept or refuse any examination.	313 (78.1)	88 (21.9)	2
**2**	When approaching the end of life, everyone has the right to accept or refuse any treatment.	316 (78.8)	85 (21.2)	1
**3**	To avoid individuals being unable to express their medical wishes in the future, they can express their wishes by signing *Palliative Care or Life-Sustaining Treatment Intention Letter*.	258 (64.3)	143 (35.7)	4
**4**	Signing the *Palliative Care or Life-Sustaining Treatment Intention Letter* indicates that the terminally ill patient can still receive medical care but no longer receive meaningless tests and treatments, such as intubation or a respirator.	267 (66.8)	133 (33.3)	3
**5**	Individuals can sign the *Palliative Care or Life-Sustaining Treatment Intention Letter* and register their wishes on their NHI card to protect their rights.	232 (57.9)	169 (42.1)	5
**@6**	The health-care power of attorney must be a spouse or blood relative.	24 (6.0)	377 (94.0)	9
**@7**	Individuals must sign an advance decision after implementing ACP consultation.	43 (10.7)	358 (89.3)	8
**8**	The instructions detailed in the *Palliative Care or Life-Sustaining Treatment Intention Letter* will be implemented only when the patient’s diseases progress to the terminal stage.	207 (51.6)	194 (48.4)	6
**@9**	Once an AD is signed, it cannot be changed or withdrawn.	114 (28.4)	287 (71.6)	7
Average total knowledge score (range 1–9 points)		4.42 (2.60)		

@ indicates the correct answer is False.

National health insurance certificate, NHI card.

### Attitudes to ACP

The mean score for elderly individuals’ attitudes was 14.32 (SD = 3.69, range 1–25). The average score for ACP benefits was 16.86 (5.62) points. The top three questions were “I believe that patients should be adequately informed about their condition so that they can prepare for the future” (18.20), “I believe that discussing ACP will inform my family or friends of my wishes and encourage them to follow them” (17.27), and “I believe that discussing ACP with my family or friends will reduce unnecessary pain at the end of my life” (17.13). The results revealed that elderly individuals agreed that appropriate truth-telling and ACP could enable them to receive the end-of-life care they want. Furthermore, the average score of each item in ACP benefits was greater than 12.5 points, indicating that elderly individuals tend to agree with the benefits of ACP described in the questionnaire.

The average score for ACP burdens was 10.82 (4.51) points. The top three questions were “I believe that discussing ACP will bring me bad luck” (11.93), “I believe that discussing ACP will make me feel hopeless” (11.62), and “I believe that discussing ACP will upset me” (10.87). The results revealed that for the burdens of ACP, elderly individuals are concerned that ACP discussion may bring bad luck and emotional distress (Table 6).

**Table 6:** Attitudes toward advance care planning (*N* = 401).

Labeling	Item	Beliefs (Bi)	Evaluations (Ei)	Attitude (AB)	Ranking
				AB = Bi*Ei	
		M (SD)	M (SD)	M (SD)	
**Benefits of ACP**		**4.06 (0.73)**	**4.00 (0.74)**	**16.86 (5.62)**	
**1**	I believe that patients should be adequately informed about their condition so that they can prepare for the future.	4.24 (0.70)	4.19 (0.76)	18.20 (5.70)	1
**2**	I believe that discussing ACP will inform my family or friends of my wishes and encourage them to follow them.	4.10 (0.81)	4.06 (0.85)	17.27 (6.27)	2
**3**	I believe that discussing ACP can reduce the burden of care on my family or friends.	4.08 (0.82)	4.03 (0.82)	17.08 (6.22)	4
**4**	I believe that discussing ACP with my family or friends will reduce unnecessary pain at the end of my life.	4.09 (0.82)	4.03 (0.83)	17.13 (6.24)	3
**5**	I believe that discussing ACP with my family or friends will help maintain my quality of life.	4.03 (0.82)	3.96 (0.87)	16.59 (6.32)	5
**7**	I believe that discussing ACP with family or friends will reduce the burden of making medical decisions for me in the future.	3.92 (0.89)	3.87 (0.87)	15.77 (6.42)	7
**8**	I believe that discussing ACP with my family or friends will reduce conflict when they make medical decisions on my behalf in the future.	3.96 (0.87)	3.91 (0.89)	16.13 (6.43)	6
**Burdens of ACP**		**3.62 (0.74)**	**2.94 (0.92)**	**10.82 (4.51)**	
**@6**	I believe that discussing ACP will upset my doctor.	3.45 (0.93)	2.86 (0.94)	10.03 (4.71)	4
**@9**	I believe that discussing ACP with my family or friends will make them uncomfortable.	3.40 (0.99)	2.80 (1.00)	9.72 (5.12)	5
**@10**	I believe that discussing ACP will upset me.	3.65 (0.92)	2.96 (1.04)	10.87 (5.17)	3
**@11**	I believe that discussing ACP will bring me bad luck.	3.82 (0.91)	3.08 (1.10)	11.93 (5.82)	1
**@12**	I believe that discussing ACP will make me feel hopeless.	3.77 (0.93)	3.03 (1.11)	11.62 (5.90)	2
Average total attitude score (range 1–25 points)				14.32 (3.69)	

### Behavioral intentions toward ACP

The mean score for behavioral intentions was 3.48 (SD = 0.85, range 1–5) points. The top three questions with the highest scores were “I will discuss my future medical-related decisions with family or friends” (3.70), “I will discuss my future medical-related decisions with health-care professionals” (3.60), and “I will sign the *Palliative Care or Life-Sustaining Treatment Intention Letter*” (3.46). Additionally, the average score of each item in the behavioral intentions toward ACP was greater than 2.5 points, indicating that elderly individuals tend to implement ACP (Table 7).

**Table 7:** Behavioral intentions toward advance care planning (*N* = 401).

Labeling	Item	M (SD)	Ranking
**1**	I will discuss my future medical-related decisions with family or friends.	3.70 (0.95)	1
**2**	I will discuss my future medical-related decisions with health-care professionals.	3.60 (0.94)	2
**3**	I will participate in an ACP consultation.	3.44 (0.99)	4
**4**	I will sign the *Palliative Care or Life-Sustaining Treatment Intention Letter*.	3.46 (0.97)	3
**5**	I will sign the health-care power of attorney form.	3.30 (1.02)	6
**6**	I will sign an advance decision through ACP consultation.	3.38 (0.99)	5
Average total behavioral intention score (range 1–5 points)		3.48 (0.85)	

### Factors affecting ACP

Factors that affect elderly individuals in implementing ACP were answered through the top three questions: “I believe that signing ADs is unnecessary because the doctor will not perform unnecessary medical procedures when I am old, such as intubation with a ventilator” (3.08), “I believe that signing ADs is unnecessary because I will make my own medical decisions when needed” (3.06), and “I believe that signing ADs is unnecessary because my family will make the best decisions for me” (3.05) (Table 8). This result indicated that trusting the doctor, the family, and oneself may prevent elderly individuals from implementing ACP.

**Table 8:** Factors affecting advance care planning (*N* = 401).

Labeling	Item	M (SD)	Ranking
**1**	I am terrified of death.	2.48 (1.00)	7
**2**	I believe that signing ADs is unnecessary because the doctor will not perform unnecessary medical procedures when I am old, such as intubation with a ventilator.	3.08 (0.96)	1
**3**	I believe that signing ADs is unnecessary because my doctor knows what I want in terms of my future care.	3.00 (0.87)	4
**4**	I believe that signing ADs is unnecessary because I will make my own medical decisions when needed.	3.06 (0.90)	2
**5**	I believe that signing ADs will disrupt divine arrangements that God (or fate or any particular deity) has for me.	2.40 (0.88)	8
**6**	I believe that I do not need to sign ADs because all suffering has a purpose.	2.87 (0.92)	5
**7**	I believe that signing ADs is only necessary for people older or sicker than me.	2.68 (0.87)	6
**8**	I believe that signing ADs is unnecessary because my family will make the best decisions for me.	3.05 (0.92)	3

### Subjective norms relating to ACP

The mean subjective norm score was 12.36 (SD = 3.79, range 1–25) points. The average score on normative belief was 3.48 (SD = 0.59, range 1–5), and the motivation to comply average score was 3.55 (0.67). “Spouse” (13.52), “nurses” (13.27), “doctors” (13.22) and “children” (13.14) were the top reference persons the study participants were willing to follow (Table 9).

**Table 9:** Subjective norms relating to advance care planning (*N* = 401).

Labeling	content	NBj	content	MCj	ΣNBj × MCj	Ranking
		M (SD)		M (SD)	M (SD)	
**1**	Do you think your spouse agrees with your views on ACP?	3.61 (0.86)	To what extent are you willing to abide by your spouse’s opinions?	3.64 (0.86)	13.52 (5.61)	1
**2**	Do you think your siblings agree with your views on ACP?	3.35 (0.81)	To what extent are you willing to abide by your siblings’ opinions?	3.37 (0.91)	11.58 (5.00)	7
**3**	Do you think your children agree with your views on ACP?	3.58 (0.78)	To what extent are you willing to abide by your children’s opinions?	3.58 (0.83)	13.14 (5.03)	4
**4**	Do you think your grandchildren agree with your views on ACP?	3.41 (0.73)	To what extent are you willing to abide by your grandchildren’s opinions?	3.39 (0.85)	11.60 (4.47)	6
**5**	Do you think your doctor agrees with your views on ACP?	3.52 (0.72)	To what extent are you willing to abide by your doctor’s opinions?	3.67 (0.72)	13.22 (4.68)	3
**6**	Do you think your nurses agree with your views on ACP?	3.54 (0.72)	To what extent are you willing to abide by nurses’ opinions?	3.66 (0.72)	13.27 (4.78)	2
**7**	Do you think your friends agree with your views on ACP?	3.39 (0.72)	To what extent are you willing to abide by your friends’ opinions?	3.41 (0.82)	11.91 (4.70)	5
Average		3.48 (0.59)		3.55 (0.67)	12.36 (3.79)	

## Discussion

The study results show that elderly individuals can learn about patient’s rights, but the current legislation in Taiwan is unclear to them. This is a common difficulty faced by countries implementing ACP in the elderly population. Official legal documents often use complicated language, making it difficult for elderly individuals to comprehend them [12]. A study in San Francisco that focused on 1400 elderly individuals found that health literacy is a strong predictor of ACP knowledge [18]. Thus, providing easy-to-understand ACP materials is paramount.

Elderly individuals agreed with the benefits of ACP described in the questionnaire, especially that appropriate truth-telling can help them prepare and receive good end-of-life care. This finding is similar to those of many studies [19–21]. A Chinese study indicated that 80.9% of elderly individuals wanted to hear the truth regarding their health condition from doctors [6]. In a qualitative study, elderly individuals wanted to know the actual status of their health and plan end-of-life care with family members to reduce the burden on the family or their suffering [19]. Therefore, notifying elderly individuals about their health conditions is crucial.

Regarding ACP burdens, elderly individuals seem to be most concerned that having an ACP discussion may bring bad luck or emotional distress. A US study of 1241 elderly individuals showed that discomfort discussing ACP was the most significant barrier to ACP implementation in the elderly population [22]. Discussing death and dying is a taboo in Asian culture, particularly Chinese culture. In this culture, it is even a taboo to use a word with the same pronunciation as “die.” For example, the pronunciation of “four” in Mandarin is similar to that of “death,” so Chinese people tend not to like things with the number four. Even the ward number of many hospitals does not have the number four. Talking about death in any way is strongly considered bad luck; people also fear that such talk may hasten death [23]. A qualitative study on Chinese-American elderly individuals found that they preferred to use indirect communication methods to discuss their preferences [24]. Therefore, health-care professionals should know how to use these indirect methods for communicating with elderly individuals and their families when implementing ACP among Chinese elderly people.

The highest scores were observed for trusting the doctor’s professionalism, the family, and trusting oneself make elderly individuals believe that signing ADs is unnecessary. Elderly individuals believe that doctors will use their expertise to make the best judgments and that family will make the best decisions for them. This finding is similar to those of many studies [25, 26]. Most East Asian countries are influenced by Confucianism and the concept of “filial piety,” and consequently, patient autonomy is considered subordinate to family values and physician authority [25, 27]. Based on this finding, raising awareness about ACP among health-care professionals and the general public can positively impact ACP promotion among elderly people. Notably, elderly individuals who believe in their own abilities and think they can make decisions when required were less likely to sign ADs. This result is similar to those of other studies that reported that preplanning is low among elderly individuals [6]. Therefore, arousing a sense of urgency for these elderly individuals is essential.

Regarding subjective norms, spouses, children, doctors, and nurses are the most crucial reference people for elderly individuals. In Chinese family-centered and collectivist culture, spouses and children are generally essential reference people [6, 26]. Not surprisingly, doctors are predominant in the patriarchal Chinese world, especially in clinical decision-making [27]. Elderly individuals in the study also considered nurses as vital reference people, which reveals that the influence of nurses should not be underestimated. Regardless of clinical settings or community care settings, nurses have more frequent contacts with elderly patients or their families than other health-care professionals. Thus, nurses can easily communicate with elderly individuals, thereby improving their understanding of ACP. A qualitative study found that because nurses are familiar with the interaction with elderly individuals and their families, they can cooperate with the healthcare team and exercise leadership in actions and interactions to defend the autonomy of elderly individuals [28]. Many studies have also reported that nurse-led or nurse-initiated ACP can improve elderly individuals’ knowledge, attitudes, chances of completing ADs, or congruence regarding the end-of-life care preferences of elderly individuals and their surrogates [29–31]. Therefore, nurses should enrich elder ACP knowledge to positively influence elderly individuals.

### Limitations

The study period coincided with the outbreak of COVID-19 around the world, including in Taiwan. We originally planned to invite seven experts to check the content validity index of the questionnaire. One expert could not provide a timely response; thus, only six conducted the CVI inspection. However, it did not affect the quality of the questionnaire. Additionally, a senior activity center where recruited the participants were closed due to the pandemic impact, resulting in imbalanced recruitment of community-dwelling elderly and elderly patients.

## Conclusions

The developed questionnaire has good validity and reliability and can help understand elderly individuals’ knowledge, attitudes, and behavioral intentions regarding ACP. Elderly individuals in the study lacked knowledge about ACP-related legislation. Although the elderly individuals agreed that ACP has certain benefits, they were worried about the emotional distress caused by ACP discussions. Spouses, children, doctors, and nurses are significant reference people for elderly individuals. Fortunately, elderly individuals with positive behavioral intentions tend to implement ACP. ACP materials or decision aids suitable for elderly individuals must be developed to increase their understanding of ACP knowledge. To enhance the general public’s awareness of ACP, ACP dialogue can be initiated in daily life through family support. The role of nurses is indispensable in promoting ACP among elderly individuals. In addition to nurse-led ACP in research, formal nurse positions such as ACP case managers can also be promoted in clinical practice.

## Supporting information

S1 File Questionnaire on knowledge, attitudes, and behavioral intentions regarding the implementation of advance care planning. (PDF)
