# Supplementary material for: Knowledge, attitudes, and behavioral intentions of elderly individuals regarding advance care planning: Questionnaire development and testing
Source: PLoS One. 2022 Jul 28;17(7):e0272351. doi: 10.1371/journal.pone.0272351 (PMC9333217; doi:10.1371/journal.pone.0272351)
Supplement: S1 File — (PDF) [file pone.0272351.s001.pdf]

Questionnaire on knowledge, attitudes, and behavioral intentions regarding the implementation of advance care planning

Knowledge of advance care planning (ACP)

- **This section seeks to identify how much you know about ACP. Please answer according to your own understanding.**

	True<br>1	False<br>2	Don't know<br>3
1. When approaching the end of life, everyone has the right to accept or refuse any examination.	☐	☐	☐
2. When approaching the end of life, everyone has the right to accept or refuse any treatment.	☐	☐	☐
3. To avoid individuals being unable to express their medical wishes in the future, they can express their wishes by signing *Palliative Care or Life-Sustaining Treatment Intention Letter*.	☐	☐	☐
4. Signing the *Palliative Care or Life-Sustaining Treatment Intention Letter* indicates that the terminally ill patient can still receive medical care but no longer receive meaningless tests and treatments, such as intubation or a respirator.	☐	☐	☐
5. Individuals can sign the *Palliative Care or Life-Sustaining Treatment Intention Letter* and register their wishes on their NHI card to protect their rights.	☐	☐	☐
6. The health-care power of attorney must be a spouse or blood relative.	☐	☐	☐
7. Individuals must sign an advance decision after implementing ACP consultation.	☐	☐	☐
8. The instructions detailed in the *Palliative Care or Life-Sustaining Treatment Intention Letter* will be implemented only when the patient's diseases progress to the terminal stage.	☐	☐	☐
9. Once an advance directive (AD) is signed, it cannot be changed or withdrawn.	☐	☐	☐

Attitudes toward ACP

- **This section aims to understand your attitudes toward ACP.**
  1. Do you agree with the following statements?
  2. How important do you think they are?

		**Agreement**					**Importance**				
		Strongly disagree to strongly agree					Not at all important to very important				
		1	2	3	4	5	1	2	3	4	5
1.	I believe that patients should be adequately informed about their condition so that they can prepare for the future.	☐	☐	☐	☐	☐	☐	☐	☐	☐	☐
2.	I believe that discussing ACP will inform my family or friends of my wishes and encourage them to follow them.	☐	☐	☐	☐	☐	☐	☐	☐	☐	☐
3.	I believe that discussing ACP can reduce the burden of care on my family or friends.	☐	☐	☐	☐	☐	☐	☐	☐	☐	☐
4.	I believe that discussing ACP with my family or friends will reduce unnecessary pain at the end of my life.	☐	☐	☐	☐	☐	☐	☐	☐	☐	☐
5.	I believe that discussing ACP with my family or friends will help maintain my quality of life.	☐	☐	☐	☐	☐	☐	☐	☐	☐	☐
6.	I believe that discussing ACP will upset my doctor.	☐	☐	☐	☐	☐	☐	☐	☐	☐	☐
7.	I believe that discussing ACP with family or friends will reduce the burden of making medical decisions for me in the future.	☐	☐	☐	☐	☐	☐	☐	☐	☐	☐
8.	I believe that discussing ACP with my family or friends will reduce conflict when they make medical decisions on my behalf in the future.	☐	☐	☐	☐	☐	☐	☐	☐	☐	☐
9.	I believe that discussing ACP with my family or friends will make them uncomfortable.	☐	☐	☐	☐	☐	☐	☐	☐	☐	☐
10.	I believe that discussing ACP will upset me.	☐	☐	☐	☐	☐	☐	☐	☐	☐	☐
11.	I believe that discussing ACP will bring me bad luck.	☐	☐	☐	☐	☐	☐	☐	☐	☐	☐
12.	I believe that discussing ACP will make me feel hopeless.	☐	☐	☐	☐	☐	☐	☐	☐	☐	☐

Behavioral intentions toward ACP

- **This section aims to understand whether you are likely to choose ACP in the future. Please answer with the first response that comes to mind.**

		**Very unlikely to very likely** 1	2	3	4	5
1.	I will discuss my future medical-related decisions with family or friends.	☐	☐	☐	☐	☐
2.	I will discuss my future medical-related decisions with health-care professionals.	☐	☐	☐	☐	☐
3.	I will participate in an ACP consultation.	☐	☐	☐	☐	☐
4.	I will sign the *Palliative Care or Life-Sustaining Treatment Intention Letter*.	☐	☐	☐	☐	☐
5.	I will sign the health-care power of attorney form.	☐	☐	☐	☐	☐
6.	I will sign an advance decision through ACP consultation.	☐	☐	☐	☐	☐

Factors affecting ACP

- **This section aims to understand the factors that affect your ACP. Please answer according to your personal situation.**

	**Strongly disagree to strongly agree** 1	2	3	4	5
1. I am terrified of death.	☐	☐	☐	☐	☐
2. I believe that signing ADs is unnecessary because the doctor will not perform unnecessary medical procedures when I am old, such as intubation with a ventilator.	☐	☐	☐	☐	☐
3. I believe that signing ADs is unnecessary because my doctor knows what I want in terms of my future care.	☐	☐	☐	☐	☐
4. I believe that signing ADs is unnecessary because I will make my own medical decisions when needed.	☐	☐	☐	☐	☐
5. I believe that signing ADs will disrupt divine arrangements that God (or fate or any particular deity) has for me.	☐	☐	☐	☐	☐
6. I believe that I do not need to sign ADs because all suffering has a purpose.	☐	☐	☐	☐	☐
7. I believe that signing ADs is only necessary for people older or sicker than me.	☐	☐	☐	☐	☐
8. I believe that signing ADs is unnecessary because my family will make the best decisions for me.	☐	☐	☐	☐	☐

Subjective norms relating to ACP

- **This section aims to understand the extent to which the people around you have influenced your ACP.**

  1. Do you think the following people would agree with your views on ACP?

  2. Are you willing to abide by their opinions?

	**Strongly disagree to strongly agree**<br>1 2 3 4 5		**Very reluctant to very willing**<br>1 2 3 4 5
1. Do you think your spouse agrees with your views on ACP?	□ □ □ □ □	To what extent are you willing to abide by your spouse's opinions?	□ □ □ □ □
2. Do you think your siblings agree with your views on ACP?	□ □ □ □ □	To what extent are you willing to abide by your siblings' opinions?	□ □ □ □ □
3. Do you think your children agree with your views on ACP?	□ □ □ □ □	To what extent are you willing to abide by your children's opinions?	□ □ □ □ □
4. Do you think your grandchildren agree with your views on ACP?	□ □ □ □ □	To what extent are you willing to abide by your grandchildren's opinions?	□ □ □ □ □
5. Do you think your doctor agrees with your views on ACP?	□ □ □ □ □	To what extent are you willing to abide by your doctor's opinions?	□ □ □ □ □
6. Do you think your nurses agree with your views on ACP?	□ □ □ □ □	To what extent are you willing to abide by nurses' opinions?	□ □ □ □ □
7. Do you think your friends agree with your views on ACP?	□ □ □ □ □	To what extent are you willing to abide by your friends' opinions?	□ □ □ □ □
